# Time-Dependent Prediction Models for Individual Prognosis of Chronic Postsurgical Pain following Knee Replacement Based on an Extensive Multivariable Data Set

**DOI:** 10.3390/jcm13030862

**Published:** 2024-02-01

**Authors:** Ulrich Betz, Michael Clarius, Manfred Krieger, Jürgen Konradi, Robert Kuchen, Lukas Schollenberger, Jörg Wiltink, Philipp Drees

**Affiliations:** 1 Institute of Physical Therapy, Prevention and Rehabilitation, University Medical Center of the Johannes Gutenberg University, 55131 Mainz, Germany; juergen.konradi@unimedizin-mainz.de; 2 Vulpius Hospital GmbH, 74906 Bad Rappenau, Germany; michael.clarius@vulpiusklinik.de; 3 Health and Care Center (GPR), 65428 Rüsselsheim, Germany; krieger@gpr-mvz.de; 4 Institute of Medical Biostatistics, Epidemiology and Informatics, University Medical Center of the Johannes Gutenberg University, 55131 Mainz, Germany; robert.kuchen@uni-mainz.de; 5 Interdisciplinary Center of Clinical Studies, University Medical Center of the Johannes Gutenberg University, 55131 Mainz, Germany; lukas.schollenberger@izks-mainz.de; 6 Department of Psychosomatic Medicine and Psychotherapy, University Medical Center of the Johannes Gutenberg University, 55131 Mainz, Germany; joerg.wiltink@unimedizin-mainz.de; 7 Department of Orthopedics and Traumatology, University Medical Center of the Johannes Gutenberg University, 55131 Mainz, Germany; philipp.drees@unimedizin-mainz.de

**Keywords:** PROMISE study, knee replacement, chronic postsurgical pain, prediction model

## Abstract

(1) Background: Clinically useful prediction models for chronic postsurgical pain (CPSP) in knee replacement (TKA) are lacking. (2) Methods: In our prospective, multicenter study, a wide-ranging set of 91 variables was collected from 933 TKA patients at eight time points up to one year after surgery. Based on this extensive data pool, simple and complex prediction models were calculated for the preoperative time point and for 6 months after surgery, using least absolute shrinkage and selection operator (LASSO) 1se and LASSO min, respectively. (3) Results: Using preoperative data only, LASSO 1se selected age, the Revised Life Orientation Test on pessimism, Western Ontario and McMaster Universities Osteoarthritis Index (WOMAC)—subscore pain and the Timed “Up and Go” Test for prediction, resulting in an area under the curve (AUC) of 0.617 and a Brier score of 0.201, expressing low predictive power only. Using data up to 6 months after surgery, LASSO 1se included preoperative Patient Health Questionnaire-4, Knee Injury and Osteoarthritis Outcome Score (KOOS)—subscore pain (pain) 3 months after surgery (month), WOMAC pain 3 and 6 months, KOOS subscore symptoms 6 months, KOOS subscore sport 6 months and KOOS subscore Quality of Life 6 months. This improved the predictive power to an intermediate one (AUC 0.755, Brier score 0.168). More complex models computed using LASSO min did little to further improve the strength of prediction. (4) Conclusions: Even using multiple variables and complex calculation methods, the possibility of individual prediction of CPSP after TKA remains limited.

## 1. Introduction

Generally, total knee arthroplasty (TKA) is a successful, safe and cost-effective treatment for improving pain and function in patients with osteoarthritis [1]. It was one of the 10 most common surgeries in Germany in 2022 [2]. The main criteria for the indication to undergo TKA are knee pain, structural damage, the failure of conservative treatment measures, a reduction in Quality of Life and subjective suffering [3]. 

Like any surgical procedure, TKA is associated with postoperative risks: 2.3% of patients develop a urinary tract infection, 1.3% develop venous thromboembolism, 0.3% develop pneumonia and 0.1% die during hospital stays [4]. Later on, with an incidence of 0.4%, periprosthetic joint infection is a feared but rather rare complication [5]. The much more common problem is chronic postsurgical pain (CPSP), which was reported in 10–34% of patients after TKA [6]. CPSP is defined as pain that develops after a surgical procedure and persists for at least 3 months after surgery [7]. Other causes of pain, such as pre-existing pain conditions or infections, or malignancy, etc., have to be excluded [8]. Pain persisting beyond the healing process after TKA is expressly included in the definition of CPSP [9] from the World Health Organization, although the surgery is performed because of an existing painful condition and chronic pain mechanisms can be active. In addition to pain intensity, CPSP also includes the dimensions of pain-related distress and pain-related interference within activities of daily living [10]. Different kinds of surgeries are associated with varying degrees of neuropathic postsurgical pain [11]. For TKA, this is reported for 6% only [12]. CPSP changes over time: from the CPSP patients in a mixed surgical cohort 6 months after surgery, 55.5% are pain-free another 6 months later [13]. The observation that the pain is sometimes not proportionate to the extent of the tissue trauma underlines the major role of individual factors [14]. Catastrophizing, mental health, preoperative knee pain and pain at other sites were found to be the strongest independent predictors of postsurgical pain in TKA [15]. In addition, a lower educational level [16], female sex and a younger age at the time of surgery [17,18], a preoperative combination of high levels of both anxiety and depressive symptoms [17,19], a higher angiotensin II type 2 receptor level [20], the presence of chronic obstructive pulmonary disease [21], diabetes mellitus or flexion contracture [22], general anesthesia [23], patellofemoral joint overstuffing [22] and postoperative coronal malalignment [24], as well as level of acute postoperative pain [25,26], have also been described as predictors of persistent pain. These numerous publications [15,16,17,18,19,20,21,22,23,24,25,26] with different predictors point to a multifactorial phenomenon. To the best of the authors’ knowledge, it has not been investigated how strong the correlation of the multiple different known predictors to CPSP is compared to each other on a large scale to date. In addition, individual factors, such as pain, function, Quality of Life, anxiety and depression, will change during the process. Previous evaluations have focused on preoperative, operative and immediate postoperative parameters, rather than on parameters later in the course.

Predictors become clinically relevant when they are used to develop predictive models that could estimate the patient’s individual risk for CPSP. In any prediction model, covariates are selected to generate an amount of information that is jointly strongly correlated with the outcome. Typically, this set of covariates includes variables that also demonstrate a robust univariate correlation with the outcome. However, it may also encompass variables that exhibit only moderate or even weak correlations with the outcome, yet contribute valuable information that complements that provided by other covariates. Primarily, a presurgical prognosis would be important, as it could lead to a reconsideration of the indication for surgery. But predictions later in the process could also be useful, as they are based on a broader database and are therefore probably more accurate. They could be used to narrow expectations and personalize the process. Such knowledge would be of great benefit for the clinical care of patients, but is currently lacking.

Palane et al. [27] have recently tested a universal predictive risk index for CPSP 12 months after TKA calculated from five variables known as predictors for their predictive power and came to the conclusion “poorly applicable”. Other predictors seem necessary to improve the predictive power. In order to determine which predictors are suitable for this, an analysis that extracts the most suitable predictors from the largest possible number of the most diverse predictors is promising. The PROMISE study (Process optimization by interdisciplinary and cross-sectoral care using the example of patients with hip and knee prostheses) [28] provides the opportunity to do this. The multicenter prospective mixed-method study gained an extensive multivariable data set collected before and in the course of up to one year after surgery. The primary endpoint of the trial was CPSP 12 months after surgery. In the supplementary subanalysis to the primary research question presented here, we investigated the relation of the numerous available parameters to CPSP and, on the basis of them, we built up different simpler and more complex models that can predict CPSP in TKA patients preoperatively and 6 months postoperatively.

In summary, CPSP after TKA is a relevant problem for which numerous predictors are known. However, it has not yet been possible to develop a clinically relevant prediction model from these. To accomplish this, useful variables were determined from a multivariable data set in the present analysis and various prediction models were developed.

## 2. Materials and Methods

### 2.1. Study Design

Three high-volume centers for arthroplasty in Germany offering different levels of care, a regional hospital, an orthopedic-specialist hospital and a tertiary referral university hospital, as well as five rehabilitation facilities, were involved in the trial. An optimized treatment standard was jointly defined and implemented at the sites equally [29]. The PROMISE process corresponds largely to interventions that are recommended by the Enhanced Recovery After Surgery (ERAS) Society for the treatment of hip and knee arthroplasty [30,31]. A wide range of sociodemographic and psychometric data were collected, along with data regarding patients’ bodies and health, symptoms, function, activity, Quality of Life, the treatment process and the surgery. For this purpose, patient-reported outcomes, medical examination findings and routinely collected data were used. Standardized test batteries were partly divided into subscores. Data were collected at the following time points: time of indication for surgery (ind), immediately preoperatively (pre), in surgery (surg), immediately postoperatively (post), at the time of discharge from rehabilitation 4–6 weeks after surgery (rehab) and 3 months (3 mo), 6 months (6 mo) and one year (1 y) after surgery. A total of 43 parameters were included in the analysis, some of which were recorded several times during treatment. Thus, a total of 91 variables were determined. An overview of the parameters collected and the corresponding time points is shown in Table 1.

Patients were eligible for study participation if they met the standardized criteria for TKA [32] and if they were able to understand the nature and extent of the study. Study exclusion criteria were as follows: patients with a life expectancy less than 1 y (e.g., those with advanced cancer), any condition that might preclude elective surgical intervention, or medical or psychological factors that would prevent them from participating or providing written informed consent. Data were recorded pseudonymously in a central electronic database and analyzed by a specialized interdisciplinary center, independent of the investigators.

### 2.2. Statistical Methods

In our study, pain is determined using the transformed WOMAC pain score [33], which is calculated from 5 questions (Question 5–9) from the KOOS [34]. To identify the score, the actual raw score was calculated and divided by the maximum achievable value of 20 points. Subsequently, the calculated score was transformed to a scale from 0 to 100, with a score of 100 indicating the complete absence of pain and 0 denoting severe pain.

In a first step, the data distributions of all considered study variables were compared between the group of patients with a good outcome, defined as WOMAC pain score > 75, and the group of patients with CPSP, defined as WOMAC pain score ≤ 75 [12]. The results were not based on the subgroup of 626 patients for whom WOMAC pain 1 y was actually observed. For each study variable, an appropriate test was performed to investigate the null hypothesis that it is equally distributed across patients with a good outcome and patients with CPSP. In the case of a continuous study variable, this corresponds to a *t*-test; in the case of a categorical variable, a chi-square test was performed, while in the case of an ordinal study variable, the Cochran–Armitage test was applied.

As is common with panel data, some of the 933 patients initially recruited dropped out during the study. However, since the probability of dropping out is likely to be correlated with some of the relevant variables, simply excluding those patients from all analyses would have been likely to result in panel attrition bias. Therefore, all following calculated univariate correlations and prediction models were determined based on 10 datasets that were created by multiple imputation, using the R package mice and predictive mean matching as the imputation method.

Subsequently, univariate Pearson correlations were determined as the mean correlation of a certain study variable, with WOMAC pain score 1 y across all imputed datasets. We use the abbreviation r in the presentation of results to denote the mean sample Pearson correlation across all ten imputed datasets. Using pooling rules for multiple imputation, standard errors for the Pearson correlations were calculated so that standard significant tests for Pearson correlation could be performed. The *p*-values of these tests quantify the evidence against the null hypothesis that a particular study variable is actually completely uncorrelated with the WOMAC pain 1 y.

Regarding prediction models, we chose to distinguish between two scenarios: one in which the pool of candidate covariates is comprised of all covariates that were available before surgery (ind and pre), and a second in which the pool of candidate covariates includes all covariates that are available 6 months after surgery. As the variable-selection method, the least absolute shrinkage and selection operator (LASSO) was used, which in addition to performing implicit variable selection, also leads to regularized estimated coefficients. LASSO is a regularization technique used in statistical modeling and machine learning to prevent overfitting and implicitly perform variable selection. The key idea behind LASSO is to add a penalty term to the standard linear regression objective function. This penalty term is proportional to the absolute values of the regression coefficients. The regularization parameter (often denoted as λ) controls the strength of the penalty. As λ increases, more coefficients are shrunk toward zero, and some may become exactly zero. LASSO can thus be implicitly used for variable selection.

Since multiple imputation was used to deal with the missing data, one would have to perform model selection not only based on one, but based on all m-imputed datasets. This, however, leads to m-prediction models, which are usually not comprised of the same set of candidate covariates. To ensure that the same candidate covariates are selected in every imputed dataset, the method of group adaptive LASSO (galasso) was used, which was proposed by Du [35] and is implemented in the R package miselect. It involves an objective function that forces the selection of the same candidate covariates across all multiply imputed datasets. A given covariate is either estimated in all datasets, or its estimated coefficients equal 0 in all datasets. The final coefficients are then obtained by averaging the estimated coefficients of a selected covariate across all imputed datasets.

It should be noted that a shrinkage parameter λ is chosen in LASSO which shrinks all coefficients towards 0 and determines the final model. The larger the value of λ, the stronger all coefficients are shrunk towards 0 and the more likely it becomes that the estimated coefficient of a certain covariate equals exactly 0, resulting in more parsimonious models. The impact of the value of λ on the predictive performances of a model is estimated by cross validation. One common choice for λ is λ_min, the value that minimizes the cross-validation criterion. The other common choice is λ_1se, the highest value of λ that leads to a cross-validation performance that is within one standard deviation of the performance obtained by λ_min. The cross validations to estimate λ_min and λ_1se depend on random fluctuations and the results can vary considerably between different runs. Therefore, these cross validations were repeated 25 times and the final values of λ_min and λ_1se were calculated by averaging the obtained values. The cross-validation performances of the two models that were obtained by λ_min (LASSO min) and λ_1se (LASSO 1se) are compared with a second outer 10-fold cross validation using the area under the curve (AUC) and the Brier score as measures for prediction accuracy.

The AUC, which corresponds to the dark gray area in Figures 3a and 4a, is the area under the receiver operating characteristic (ROC) curve. The ROC curve, which corresponds to the thick black line in Figures 3a and 4a, is a graphical representation of the true positive rate (sensitivity) against the false positive rate (1-specificity) for different threshold values. Each point on the ROC curve represents a sensitivity and specificity pair corresponding to a particular decision threshold. The AUC provides a single value that summarizes the overall performance of a classification model across various threshold values. It ranges from 0 to 1, with a higher value indicating better discrimination between the positive and negative classes.

The Brier score is a metric used to assess the accuracy of probabilistic predictions in a binary classification setting. It measures the mean squared difference between predicted probabilities and the actual outcomes (non-event: 0, event: 1) for each instance in the dataset.

**Figure 1:**
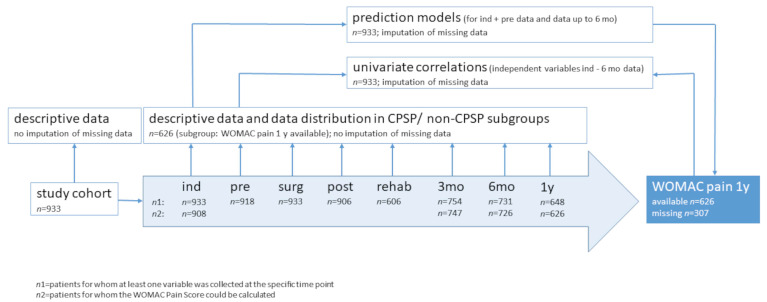
CONSORT flow-chart and analysis methodology.

To make the effect sizes of the selected covariates comparable, the same prediction model was not only estimated based on the original, but also based on standardized data. For the CONSORT flow-chart supplemented by the representation of the analysis methodology, see Figure 1.

All statistical analyses were conducted in R. For multiple imputations, the mice package was utilized. Variable selection based on the multiply imputed datasets was carried out using the miselect package, which implements the GALASSO method in the homonymous function. The optimal value of the shrinkage parameter was estimated using the cv.galasso function from the same package.

## 3. Results

### 3.1. Study Subjects

Our analysis included 933 study participants receiving TKA: 55.4% were female and 50.7% had undergone right-knee surgery. The mean age was 67.1 years (SD 9.6), mean BMI was 30.2 (SD 5.8), and mean ASA was 2.3 (SD 0.6). Patients had an average of 1.7 (SD 1.4) comorbidities. Important patients’ baseline characteristics are shown in Table 2.

### 3.2. Pain Profile during the Process

**Figure 2:**
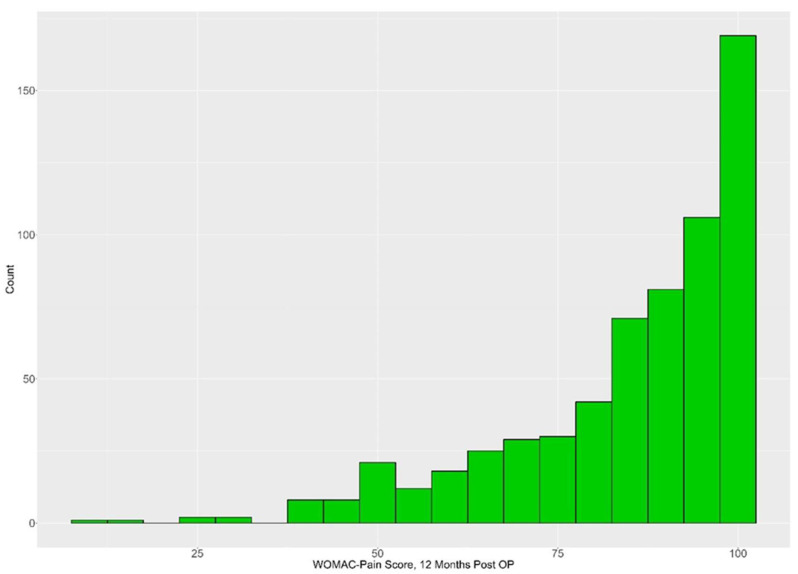
Histogram for WOMAC pain score results 1 year after TKA.

Considering all available data, with the number of participants decreasing over time, the results for the different measurement time points are shown in Table 3. The mean WOMAC score increased from 50.84 at baseline to 84.93 one year after surgery. For the 620 patients for whom the WOMAC pain score was available at baseline and at 1-year follow-up, the pain scores improved by an average of 32.92 (SD 21.57; median 35.00; Q1, Q3: 20.00, 50.00; range −40–95) points. The vast majority of patients (74.9%) achieved 80 or more points in the WOMAC pain score one year after the surgery (Figure 2).

### 3.3. Data Distributions between Subgroups with/without CPSP

For a subgroup of 626 subjects, the transformed WOMAC pain score 1 y is available. For this subgroup, the results of the 91 variables examined in our project were determined and presented in total and separately in subgroups with WOMAC pain after 1 y ≤ 75 or >75 points. The descriptive results for those covariates whose distributions were significantly different between the two groups are shown in Table 4. The complete presentation of the results, including all variables and even more descriptive data, can be found in the Supplementary Materials (see Table S1). Overall, subgroup results differed significantly (*p* < 0.05) in 47 variables. This includes variables recorded at all measurement points up to 6 months after surgery and for all test categories, excluding only the process and surgery category.

### 3.4. Univariate Correlations

For an overview of the strength of the univariate relationship between the different parameters at the different time points and the WOMAC pain 1 y in our sample, see Table 5. The strongest correlations were found for KOOS pain (r = 0.56), WOMAC pain (r = 0.55) and the KOOS ADL (r = 0.55) 6 months after surgery. These variables showed a slightly lower correlation 3 months after surgery (KOOS ADL r = 0.51; WOMAC pain r = 0.48; KOOS pain r = 0.48). Thus, 3 months after surgery they were on a level with the QoL parameters KOOS QoL (r = 0.47) and EQ-5D-5L (r = 0.45), as well as the activity parameter KOOS sport (r = 0.47) after 6 months. In total, the strength of the relationship was close at 3 and 6 months postoperatively, e.g., KOOS symp after 3 months r = 0.43 and after 6 months r = 0.47 or SSD Q2 after 3 months r = 0.41 and after 6 months r = 0.42. PHQ-4 score was highest on our psychometric screening tools 6 months after surgery (r = −0.38). PHQ-4 is also the strongest predictor at the end of rehabilitation (r = 0.32) and before surgery (r = 0.29). Thus, higher PHQ-4 values are obtained after rehabilitation or even preoperatively than, for example, for pain measurement using VAS/NRS at rest (pre r = −0.20; rehab r = −0.23). In the various categories, the strongest predictors are as follows (see Table 5): ISAR score preoperatively (health category; r = −0.16), age at the time of the surgery (sociodemographic data category; r = 0.12), patients’ weight at surgery (body characterization category; r = 0.10), TUG post (function category; r = −0.10), duration of the surgery (surgery category; r = −0.06) and LOS (process category; r = −0.04).

### 3.5. Prediction Models

**Figure 3:**
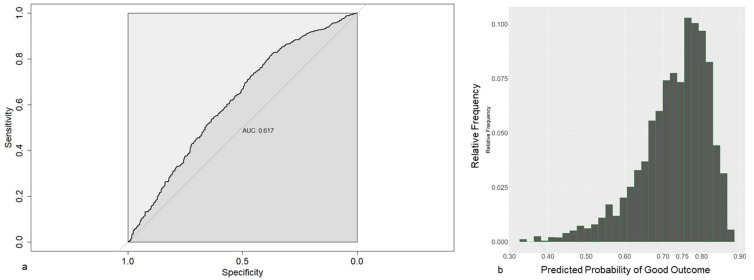
(**a**) Receiver operating characteristics for prediction models based on preoperative data, using LASSO-1se. (**b**) Frequencies of the estimated probabilities for the prediction model based on preoperative data.

From the available data pool, time-dependent prediction models could be calculated for the risk of WOMAC pain 1 y ≤ 75, defined as CPSP in our trial. The LASSO-1se model, obtained using all covariates available before surgery as candidate covariates, is shown in Table 6, once based on the original data, and once based on standardized data. The model contains the covariates age, LOT-R pess, WOMAC pain and TUG. The predictive performances of the model were rather weak, illustrated by the relatively high Brier score (0.201), the relatively low AUC (0.617, Figure 3a) and the fact that most predictions are closely clustered around 0.75 (Figure 3b), which is approximately the proportion of patients with a good outcome 1 year after surgery.

In contrast, the LASSO-1se model obtained, using all covariates available 6 months after surgery as candidate covariates, included seven covariates, PHQ-4 score pre, KOOS pain 3 mo, WOMAC pain 3 mo, KOOS symp 6 mo, KOOS sport 6 mo, KOOS QoL 6 mo and WOMAC pain 6 mo (Table 7).

**Figure 4:**
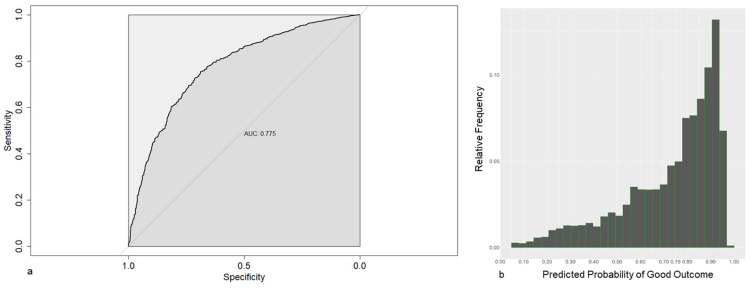
(**a**) Receiver operating characteristic for a prediction model based on 6 months post surgery data, using LASSO-1se. (**b**) Frequencies of the estimated probabilities for the prediction model based on 6 months post surgery data, using LASSO-1se.

The predictive performances (as estimated by cross-validation) to distinguish between patients with CPSP and Non-CPSP tend to be much better, as shown by the Brier score (0.168) and the AUC (0.755, Figure 4a). This becomes apparent when looking at its predicted probabilities (Figure 4b), which are now substantially more scattered across the probability space.

Using LASSO min for modeling, more variables are included (model with preoperative data: 16 variables; model with data up to 6 months after surgery: 26 variables). Both models are described in the Supplementary Materials (see Tables S2 and S3). With these complex models, we obtained an AUC of 0.634 and a Brier score of 0.199 (model with preoperative data), and an AUC of 0.774 and a Brier score of 0.17 (model with data up to 6 months after surgery). Thus, the very complex models did not improve the predictive power much, if at all, compared to the simpler models.

## 4. Discussion

The Minimum Clinically Important Difference for improvement in WOMAC pain score was exceeded by far in this trial [36], but a not-negligible number of the subjects developed a CPSP. In general, data on CPSP are subject to wide variability due to different definitions and methodological differences in data collection [37] and are therefore difficult to compare. Regardless, the median incidence of chronic pain 6–12 months after surgery is specified at 20–30%, with a slight decrease over time [38]. With this in mind, the incidence of chronic pain after TKA is comparable to that after other surgeries [2] and the data obtained in this study fit seamlessly with the findings reported for TKA in the current literature [39]. Because TKA is an elective procedure with which patients primarily associate the hope of pain relief [40], it is conceivable that a CPSP after TKA may be perceived as particularly disappointing and distressing compared to other conditions.

Multiple differences in the preoperative demographic and clinical characteristic variables between long-term satisfied and dissatisfied patients after TKA have already been identified [41,42]. This is confirmed in our cohort regarding CPSP. We found significant differences between subgroups with a good outcome and with CPSP in the different categories and at different time points.

Accordingly, the data analysis of this study was able to establish numerous univariate correlations between the variables at various time points in the treatment process and pain one year after surgery. To the best of the authors’ knowledge, this provided the possibility of comparing time-dependent parameters in terms of their strength on a large scale for the first time. With r > 0.5, strong correlations could be determined for various parameters 6 months postoperatively. The result that we did not find even stronger associations between parameters at 6 months and WOMAC pain 1 y, even with the WOMAC pain score at 6 months as the independent variable and at 1 y as the dependent variable, indicates that changes in pain perception are still possible after 6 months. However, as a subjectively perceived phenomenon, pain always remains difficult to measure, so very strong correlations may not be expected at all. The fact that, in addition to the pain scores, the KOOS ADL also showed a comparably high correlation at this time may be due to the fact that the existing pain has a direct effect on ADL competence, as described for physical activity [43]. The relatively strong correlation values for the QoL and activity parameters underline this consideration, but could not be proven with univariate correlations. Well-known and already used in therapy [44] is the effect of pain on people’s psychological situation [45]. This could be the reason for the high correlation values of the PHQ-4 measurements at different time points of the treatment. Interestingly, mental distress (PHQ-4) in the early phase of the process was a stronger predictor than pain. This underlines the relevance of mental conditions like distress, but also confidence in the treatment for the long-term success of treatments. Wunderlich et al. [46] have described this relationship for the function accordingly after TKA. Overall, the correlation of the variables in the preoperative and immediate postoperative period to CPSP are rather weak. Thus, pain measurements, such as the WOMAC pain score, achieve correlation values of about r = 0.2 in this phase, although preoperative pain is mentioned as one of the strongest independent predictors of CPSP after TKA in the review by Lewis [14]. Moreover, the current literature directly cites postoperative pain as a predictor [25,26]. Also, contrary to the literature [23] is our finding that general anesthesia has no correlation to CPSP in our cohort, just as all other data we collected on surgery. Regarding sociodemographic parameters, significant correlations in our data could only be determined for income and age, and this at a very low level. As stated in the literature for TKA [17,18] and other surgeries [47], our younger patients were at higher risk. However, the sociodemographic factors in CPSP were not very clear. This includes the gender of the patient, although the literature indicates that women are at higher risk [17,18]. The situation in our health category is comparable. Here, only the ISAR had a correlation to CPSP, and that was at a very low level. For all other parameters in this category, we found no correlation in our data, with the same being true for smoking, although such a correlation has been described for other pain problems [48]. Regarding the parameters describing our process, we did not identify any correlations with the CPSP in our data, either. Information on this is still missing in the literature.

It is possible to create predictive models from the available demographic and clinical characteristic variables. In our trial, however, the excellent predictive capabilities as described for dissatisfaction after one year [49] could not be confirmed. If only preoperative data are included, the predictive power for CPSP is low, not only with a clinically feasible model with a strongly reduced number of variables, but also with a more complex model that includes a higher number of covariates. Only expanding the data up to 6 months after surgery improves the strength of the predictive models to an intermediate level. Again, the reduced and the complex model remained comparable in their predictive power. It is important to note that the inclusion of variables in the prediction models is not solely based on the strength of the correlation in the univariate analysis. Rather, the procedure aims to determine the best possible aggregation of information to predict CPSP. Variables that are not strongly correlated with the outcome can also be included in the model. The value of individual univariate correlations should therefore not be overestimated.

The strength of the present prospective, multicenter study is the wide range of data collection at different time points up to one year after surgery, with a good number of patients. As a result, a large number of variables could be determined in which CPSP and non-CPSP subgroups are significantly different and correlate with CPSP. On this broad basis, different time-dependent prediction models for CPSP after TKA could be developed.

The analysis also has some limitations. Not all categories considered as causes of CPSP in the literature were included. Thus, serologic and radiologic analyses were not performed, although there is evidence in the literature that predictors of CPSP can be found there as well [9,11]. However, it is not expected that a hidden “top parameter” exists that can reliably predict CPSP, as this would likely be very closely linked to the pain, function, and activity data which have already been collected. It remains unclear whether it is possible and, if so, which parameters could be used to develop a prediction model with high predictive power. Another limitation is missing data. A total of 933 patients were enrolled in the study at the time of the surgical indication, and all these patients underwent surgery. Among them, 15 patients (1.6%) did not attend the immediate preoperative examination. As is usual in observational studies, an increasing number of patients dropped out over the follow-up period, resulting in only 626 patients who reported their pain level one year after surgery. It is important to note that, of the initially included 933 patients, all missing values, both before and after surgery, were imputed using the gold-standard method of multiple imputation. Since multiple imputation is recognized for providing relatively accurate results, even in the presence of a non-random drop-out mechanism, we believe that the impact of missing data on the results is minimal.

## 5. Summary

A comprehensive data set with numerous variables was analyzed at different time points before and after surgery. For the examined cohort 12 months after TKA, CPSP occurred to an extent known from the literature. A large number of significant differences in the data distribution between the patients with and without CPSP and numerous univariate correlations between the variables at various time points in the treatment process and pain one year after surgery were found. LASSO was used to select the most appropriate variables for different prediction models from the available pool and to estimate regularized coefficients. Different shrinkage parameters were used to fit simpler and more complex models for the preoperative time point and the time point 6 months after surgery. Despite the large number of available variables and even with complex models, the predictive power of the models is only low preoperatively and intermediate 6 months after surgery. The intended clinical application of the models will thus hardly be possible in a meaningful way. The indication must continue to be made on the basis of the valid indication criteria, without being able to take a possible increased risk of CPSP into account. In addition, it is still not possible to narrow patient expectations and personalize treatment on the basis of a prediction model for CPSP.

## 6. Conclusions

It is possible to build time-dependent predictive models, and both simple and complex models, allowing the prediction of CPSP with only low strength at an early stage. Later in the course of treatment, the predictive models gain strength without achieving high predictive power, even with a very large number of variables and very complex models. The value of the models for clinical practice therefore remains limited.

## Figures and Tables

**Table 1:** Parameters and data collection time points.

Parameter	Short Form *	Category	Values	Time Point							
				ind	pre	surg	post	rehab	3 mo	6 mo	1 y
**Knee Injury and Osteoarthritis Outcome Score (KOOS)—subscore sport** Questionnaire to assess short- and long-term patient-relevant outcomes following knee injury. Five subscores: pain, symptoms, Activities of Daily Living, sport and recreation function and knee-related Quality of Life; self-administered	KOOS sport	Activity	Score range 0–100	X					X	X	X
**Knee Injury and Osteoarthritis Outcome Score (KOOS)—subscore ADL**	KOOS ADL	ADL	Score range 0–100	X					X	X	X
**Somatic Symptom Disorder (SSD) Q2: During the last 7 days, my bodily symptoms interfered with daily life activities** Two-item screening questionnaire to assess somatic symptom disorder.	SSD Q2	ADL	Score range 0–10	X	X		X	X	X	X	X
**Height of the patient**	Height	Body characterization	Centimeters			X					
**Weight at day of surgery**	Weight	Body characterization	Kilograms			X					
**Length of hospital stay**	LOS	Function	Days				X				
**Possible walking distance**	Walking dist	Function	Score range 1–5				X				
**Timed “Up and Go” Test**	TUG	Function	Seconds		X		X	X			
**Alcohol consumption on a regular basis**	Alcohol	Health	Yes/no	X							
**American Society of Anesthesiologists (ASA) classification** Grading system for a person’s state of health before a surgical procedure.	ASA	Health	Score range 1–6		X						
**Identification of Seniors at Risk (ISAR)** Questionnaire to identify seniors with increased risk of adverse functional outcomes.	ISAR	Health	Score range 0–6		X						
**Smoker**	Smoker	Health	Yes/no	X							
**Did the patient participate in the preoperative patient school?**	Patients School	Process	Yes/no				X				
**Duration of rehabilitation**	Rehab duration	Process	Days					X			
**Duration of pre-treatment**	Pre-treatment	Process	Days	X							
**Life Orientation Tests (LOT-R) on optimism** Questionnaire to assess the dispositional level of optimism/pessimism.	LOT-R opt	Psychometric	Score range 0–12	X							
**Life Orientation Tests (LOT-R) on pessimism** Questionnaire to assess the dispositional level of optimism/pessimism.	LOT-R pess	Psychometric	Score range 0–12	X							
**Oslo Social Support Scale (OSSS)** Three-item **screening questionnaire** to assess perceived social support.	OSSS	Psychometric	Score range 3–14	X							
**Patient Health Questionnaire-4 (PHQ-4)** Four-item **screening** questionnaire to assess mental distress (anxiety/depression).	PHQ-4	Psychometric	Score range 0–12	X	X		X	X	X	X	X
**European Quality of Life 5 Dimensions 5 Level Version (EQ-5D-5L)** Questionnaire for measuring patient-reported outcomes to assess the Quality of Life of patients regardless of their illness.	EQ-5D-5L	QoL	Score range < 0–1	X			X	X	X	X	X
**Knee Injury and Osteoarthritis Outcome Score (KOOS)—subscore QoL**	KOOS QoL	QoL	Score range 0–100	X					X	X	X
**Age at day of surgery**	Age	Sociodemographic	Years	X							
**Higher school degree**	Higher school	Sociodemographic	Yes/no	X							
**Marital status—married or single with partner**	Marital with	Sociodemographic	Yes/no	X							
**Number of children**	Number children	Sociodemographic	Number	X							
**Female Sex**	Female sex	Sociodemographic	Yes/male	X							
**What is the total monthly net income of your household in euros?**	Income	Sociodemographic	Score range 1–8	X							
**Employment situation—full or part-time (15–34 h/week) employed**	Employed	Sociodemographic	Yes/others	X							
**Cortisone application preoperatively**	Cortisone	Surgery	Yes/no			X					
**Duration of the surgery**	Surg duration	Surgery	Minutes			X					
**Tranexamic acid application intraoperatively**	Surg tranex	Surgery	Yes/no			X					
**Intubation anesthesia**	Intub anesthesia	Surgery	Yes/spinal anesthesia			x					
**Use of local infiltration analgesia (LIA)**	Surg LIA	Surgery	Yes/no			x					
**Use of pneumatic tourniquet**	Surg tourn	Surgery	Yes/no			x					
**Use of drains**	Surg drain	Surgery	Yes/no			x					
**Knee Injury and Osteoarthritis Outcome Score (KOOS)—subscore pain**	KOOS pain	Symptoms	Score range 0–100	X					X	X	X
**Knee Injury and Osteoarthritis Outcome Score (KOOS)—subscore symptoms**	KOOS symp	Symptoms	Score range 0–100	X					X	X	X
**SSD Q1 During the last 7 days, the overall intensity of my bodily symptoms was** Two-item screening questionnaire to assess somatic symptom disorder.	SSD Q1	Symptoms	Score range 0–10	X	X		X	X	X	X	X
**Visual analog scale (VAS)/Numeric Rating scale (NRS) at rest**	Pain rest	Symptoms	Score range 0–10		X		X	X			
**Visual analog scale (VAS)/Numeric Rating scale (NRS) with load**	Pain load	Symptoms	Score range 0–10		X		X	X			
**Western Ontario and McMaster Universities Osteoarthritis Index (WOMAC)—subscore pain** Questionnaire to evaluate the condition of patients with osteoarthritis of the knee and hip, including pain, stiffness and physical functioning of the joints.	WOMAC pain	Symptoms	Score range 0–20	X					X	X	X
**Staffelstein Score** Questionnaire to assess rehabilitation success after hip and knee arthroplasty, including pain, activities of daily living and physical functioning of the joints.	Staffelstein	Symptoms/ADL/Function	Score range 0–120	X			X	X			

* The parameter short forms are used in the text below. Abbreviations: Time of indication for surgery (ind), immediately preoperatively (pre), in surgery (surg), immediately postoperatively (post), at the time of discharge from rehabilitation 4–6 weeks after surgery (rehab), 3 months (3 mo), 6 months (6 mo) and 1 year (1 y) after surgery.

**Table 2:** Patient characteristics.

		TKA (*n* = 933)
	Missing	*n* (%)
Sex		
male		416 (44.6)
female		517 (55.4)
Joint		
knee left		490 (49.3)
knee right		504 (50.7)
Comorbidities		
at least one comorbidity		744 (79.7%)
		mean (SD)
number of comorbidities		1.7 (1.4)
ASA	77	2.3 (0.6)
Age	52	67.1 (9.6)
BMI	73	30.2 (5.8)

**Table 3:** Details for transformed WOMAC pain scores at different time points of the process.

Visit	*n*	Minimum	1st Quartile	Median	Mean	3rd Quartile	Maximum
ind	908	0	40	50	50.84	65	100
3 mo	747	0	60	75	74.20	90	100
6 mo	726	20	70	85	79.73	95	100
1 y	626	10	76.25	90	84.93	100	100

**Table 4:** Descriptive results for variables with a significant difference (*p* < 0.05) between the subgroups. The values in columns 3–5 indicate mean (SD).

Parameter Short Form	Time Point	WOMAC Pain ≤75 (*n* = 157)	WOMAC Pain >75 (*n* = 469)	Total (*n* = 626)	*p* Value
Age	ind				0.002
		65.06 (9.79)	67.62 (8.74)	66.97 (9.08)	
SSD Q1	ind				0.013
		6.20 (1.73)	5.74 (1.94)	5.86 (1.90)	
Income	ind				0.032
		5.15 (2.00)	5.60 (1.94)	5.49 (1.97)	
EQ-5D-5L	ind				0.011
		0.63 (0.19)	0.67 (0.18)	0.66 (0.18)	
OSSS	ind				<0.001
		10.51 (2.21)	11.17 (1.96)	11.00 (2.04)	
PHQ-4	ind				<0.001
		3.49 (2.96)	2.14 (2.28)	2.48 (2.53)	
LOT-R opt	ind				<0.001
		9.45 (2.42)	10.46 (2.18)	10.21 (2.28)	
LOT-R pess	ind				<0.001
		8.23 (3.06)	9.70 (2.59)	9.33 (2.79)	
KOOS pain	ind				<0.001
		41.90 (15.72)	48.13 (17.28)	46.56 (17.10)	
KOOS symp	ind				<0.001
		47.33 (17.55)	55.73 (19.38)	53.62 (19.28)	
KOOS ADL	ind				<0.001
		50.54 (19.24)	57.06 (17.93)	55.42 (18.47)	
Staffelstein	ind				0.010
		78.33 (12.43)	81.55 (12.37)	80.77 (12.45)	
WOMAC pain	ind				<0.001
		45.80 (17.12)	53.99 (18.29)	51.93 (18.33)	
Pain rest	pre				<0.001
		3.48 (2.44)	2.43 (2.37)	2.68 (2.43)	
Pain load	pre				0.016
		5.25 (2.30)	4.64 (2.63)	4.79 (2.56)	
ISAR	pre				0.005
		0.58 (0.84)	0.35 (0.72)	0.41 (0.76)	
PHQ-4	pre				<0.001
		3.81 (3.17)	2.03 (2.13)	2.43 (2.52)	
Weight	surg				0.027
		90.55 (18.46)	86.90 (17.76)	87.81 (18.00)	
TUG	post				0.023
		18.22 (8.26)	16.70 (5.91)	17.09 (6.62)	
Pain rest	post				<0.001
		2.62 (1.90)	1.92 (1.72)	2.10 (1.79)	
Pain load	post				0.002
		3.89 (1.95)	3.32 (1.80)	3.46 (1.85)	
EQ-5D-5L	post				0.022
		0.76 (0.17)	0.80 (0.15)	0.79 (0.15)	
PHQ-4	post				<0.001
		2.71 (2.79)	1.71 (2.10)	1.96 (2.33)	
EQ-5D-5L	rehab				<0.001
		0.77 (0.14)	0.84 (0.11)	0.82 (0.12)	
Pain rest	rehab				0.001
		2.28 (1.85)	1.50 (1.51)	1.72 (1.65)	
Pain load	rehab				0.002
		3.68 (1.94)	2.86 (1.71)	3.09 (1.81)	
PHQ-4	rehab				<0.001
		2.49 (2.12)	1.04 (1.73)	1.42 (1.95)	
Staffelstein	rehab				<0.001
		85.04 (12.36)	92.43 (13.21)	90.73 (13.37)	
SSD Q1	3 mo				<0.001
		4.21 (1.88)	2.72 (1.97)	3.10 (2.05)	
EQ-5D-5L	3 mo				<0.001
		0.74 (0.18)	0.87 (0.11)	0.84 (0.14)	
KOOS pain	3 mo				<0.001
		55.16 (17.13)	75.76 (15.37)	70.61 (18.16)	
KOOS symp	3 mo				<0.001
		54.06 (17.52)	72.40 (15.05)	67.83 (17.58)	
KOOS ADL	3 mo				<0.001
		60.62 (16.21)	79.64 (13.60)	74.88 (16.49)	
KOOS sport	3 mo				<0.001
		29.41 (21.90)	49.59 (25.18)	44.46 (25.91)	
KOOS QoL	3 mo				<0.001
		36.53 (19.02)	56.66 (20.86)	51.56 (22.19)	
WOMAC pain	3 mo				<0.001
		59.90 (17.48)	79.86 (14.38)	74.87 (17.49)	
PHQ-4	3 mo				<0.001
		2.97 (2.86)	1.09 (1.90)	1.57 (2.33)	
SSD Q1	6 mo				<0.001
		4.16 (1.95)	2.24 (1.85)	2.71 (2.05)	
SSD Q2	6 mo				<0.001
		4.13 (2.14)	2.08 (1.93)	2.59 (2.17)	
EQ-5D-5L	6 mo				<0.001
		0.75 (0.18)	0.90 (0.11)	0.86 (0.14)	
PHQ-4	6 mo				<0.001
		2.88 (2.66)	0.93 (1.64)	1.40 (2.11)	
KOOS pain	6 mo				<0.001
		58.34 (16.85)	82.80 (13.86)	76.96 (17.95)	
KOOS symp	6 mo				<0.001
		58.21 (14.42)	78.81 (14.61)	73.91 (17.00)	
KOOS ADL	6 mo				<0.001
		62.72 (15.65)	84.16 (12.55)	79.11 (16.15)	
KOOS sport	6 mo				<0.001
		32.29 (21.60)	58.92 (23.32)	52.53 (25.57)	
KOOS QoL	6 mo				<0.001
		40.65 (19.52)	65.57 (20.48)	59.59 (22.87)	
WOMAC pain	6 mo				<0.001
		63.05 (17.17)	86.01 (12.99)	80.53 (17.16)	
SSD Q1	1 y				<0.001
		3.95 (2.07)	1.84 (1.81)	2.36 (2.09)	
SSD Q2	1 y				<0.001
		4.03 (2.28)	1.64 (1.80)	2.24 (2.19)	
EQ-5D-5L	1 y				<0.001
		0.75 (0.18)	0.93 (0.10)	0.89 (0.14)	
PHQ-4	1 y				<0.001
		2.74 (2.99)	0.80 (1.49)	1.29 (2.15)	
KOOS symp	1 y				<0.001
		61.51 (15.61)	84.79 (12.11)	79.01 (16.48)	
KOOS ADL	1 y				<0.001
		62.81 (16.35)	90.22 (9.15)	83.32 (16.47)	
KOOS sport	1 y				<0.001
		35.57 (24.58)	67.68 (21.08)	59.42 (26.11)	
KOOS QoL	1 y				<0.001
		42.31 (18.54)	72.95 (18.71)	65.26 (22.90)	

**Table 5:** Checked parameters at the time points, sorted according to the magnitude of the correlation = r. The abbreviation r denotes the mean sample Pearson correlation across all ten imputed datasets. For short-form description, see Table 1.

Parameter—Short Form	Time Point	r	*p*
KOOS pain	6 mo	0.555	<0.0001
WOMAC pain	6 mo	0.554	<0.0001
KOOS ADL	6 mo	0.550	<0.0001
KOOS ADL	3 mo	0.510	<0.0001
WOMAC pain	3 mo	0.483	<0.0001
KOOS pain	3 mo	0.480	<0.0001
KOOS symp	6 mo	0.474	<0.0001
KOOS QoL	6 mo	0.467	<0.0001
KOOS sport	6 mo	0.466	<0.0001
EQ-5D-5L	6 mo	0.445	<0.0001
KOOS symp	3 mo	0.427	<0.0001
SSD Q1	6 mo	−0.419	<0.0001
EQ-5D-5L	3 mo	0.418	<0.0001
SSD Q2	6 mo	−0.416	<0.0001
SSD Q2	3 mo	−0.407	<0.0001
KOOS QoL	3 mo	0.407	<0.0001
KOOS sport	3 mo	0.385	<0.0001
PHQ-4	6 mo	−0.383	<0.0001
SSD Q1	3 mo	−0.369	<0.0001
PHQ-4	3 mo	−0.363	<0.0001
PHQ-4	rehab	−0.320	<0.0001
PHQ-4	pre	−0.294	<0.0001
EQ-5D-5L	rehab	0.284	<0.0001
Staffelstein	rehab	0.269	<0.0001
LOT-R pess	ind	0.251	<0.0001
PHQ-4	ind	−0.249	<0.0001
Pain load	rehab	−0.235	<0.0001
SSD Q2	rehab	−0.235	<0.0001
Pain rest	rehab	−0.225	0.0002
KOOS ADL	ind	0.217	<0.0001
SSD Q1	rehab	−0.215	<0.0001
WOMAC pain	ind	0.214	<0.0001
PHQ-4	post	−0.205	<0.0001
Pain rest	pre	−0.200	<0.0001
Pain rest	post	−0.191	<0.0001
KOOS pain	ind	0.187	<0.0001
LOT-R opt	ind	0.184	<0.0001
EQ-5D-5L	ind	0.180	<0.0001
KOOS symp	ind	0.171	<0.0001
Pain load	pre	−0.157	0.0001
Pain load	post	−0.156	0.0005
ISAR	pre	−0.155	<0.0001
KOOS sport	ind	0.151	<0.0001
OSSS	ind	0.148	0.0001
Staffelstein	ind	0.142	0.0004
EQ-5D-5L	post	0.136	0.0006
SSD Q1	ind	−0.129	0.0009
KOOS QoL	ind	0.125	0.0008
Age	ind	0.124	0.001
SSD Q2	pre	−0.120	0.001
SSD Q1	pre	−0.111	0.005
Staffelstein	post	0.109	0.004
Weight	surg	−0.104	0.008
Income	ind	0.104	0.010
TUG	post	−0.103	0.022
SSD Q2	ind	−0.102	0.006
TUG	pre	−0.098	0.015
TUG	rehab	−0.087	0.121
SSD Q2	post	−0.078	0.037
SSD Q1	post	−0.076	0.053
Surg duration	surg	−0.059	0.118
Intub anesthesia	surg	−0.056	0.185
Number children	ind	0.049	0.194
LOS	post	−0.044	0.304
Surg tourn	surg	0.042	0.268
Alcohol	ind	0.041	0.289
Surg LIA	surg	0.036	0.406
Smoker	ind	−0.035	0.357
Cortisone	surg	−0.035	0.364
Higher school	ind	0.030	0.397
Walking dist	post	0.029	0.595
Patients school	post	0.025	0.505
Rehab duration	rehab	−0.018	0.711
Martial with	ind	0.018	0.648
Female sex	ind	−0.016	0.673
Employed	ind	−0.016	0.684
Time till surg	pre	0.014	0.722
Surg drain	surg	−0.013	0.724
Height	surg	0.010	0.796
Surg tranex	surg	0.005	0.899
ASA	pre	−0.004	0.915

**Table 6:** Details for the prediction model based on preoperative data, non-standardized and standardized, using LASSO-1se.

Using Non-Standardized Data				
(Intercept)	Age, ind	LOT-R pess, ind	WOMAC pain, ind	TUG, pre
−1.1000	0.0100	0.1100	0.0089	−0.0004
**Using Standardized Data**				
(Intercept)	Age, ind	LOT-R pess, ind	WOMAC pain, ind	TUG, pre
1.005	0.097	0.316	0.165	−0.002

**Table 7:** Details for the prediction model based on all data until 6 months post surgery, non-standardized and standardized, using LASSO-1se.

Using Non-Standardized Data			
(Intercept)	PHQ-4 Score, pre	KOOS pain, 3 mo	WOMAC pain, 3 mo
−3.784	−0.088	0.008	0.014
KOOS symp, 6 mo	KOOS sport, 6 mo	KOOS QoL, 6 mo	WOMAC pain, 6 mo
0.019	0.007	0.002	0.023
**Using Standardized Data**			
(Intercept)	PHQ-4 Score, pre	KOOS pain, 3 mo	WOMAC pain, 3 mo
1.207	−0.224	0.144	0.253
KOOS symp, 6 mo	KOOS sport, 6 mo	KOOS QoL, 6 mo	WOMAC pain, 6 mo
0.335	0.179	0.040	0.400

## Data Availability

For additional information, the results report of the PROMISE project can be found at: https://innovationsfonds.g-ba.de/beschluesse/promise-prozessoptimierung-durch-interdisziplinaere-sektorenuebergreifende-versorgung-am-beispiel-von-hueft-und-kniearthrosen.120 (accessed on 12 October 2023). Further sub-analyses of the data pool have been published in scientific journals. The underlying data have not been released for publication because this is not covered by the given ethics vote.

## Supplementary Materials

The following supporting information can be downloaded at: https://www.mdpi.com/article/10.3390/jcm13030862/s1, Table S1: Extended descriptive data for all variables; Table S2: Details for the prediction model based on preoperative data, non-standardized and standardized, using LASSO min; Table S3: Details for the prediction model based on data up to 6 months after surgery, non-standardized and standardized, using LASSO min.
