# Supplementary material for: Time-Dependent Prediction Models for Individual Prognosis of Chronic Postsurgical Pain following Knee Replacement Based on an Extensive Multivariable Data Set
Source: J Clin Med. 2024 Feb 1;13(3):862. doi: 10.3390/jcm13030862 (PMC10856264; doi:10.3390/jcm13030862)
Supplement: Supplementary file 1 [file jcm-13-00862-s001.zip › jcm-2775304-supplementary (1).pdf]

Table S1: Extended descriptive data for all variables

Parameter - short form	Time point	WOMAC pain ≤75 (N=157)	WOMAC pain >75 (N=469)	Total (N=626)	p value
**Sex**	ind				0.581
**Male**		72 (45.9%)	227 (48.4%)	299 (47.8%)	
**Female**		85 (54.1%)	242 (51.6%)	327 (52.2%)	
**Smoker**	ind				0.850
**N-Miss**		0	3	3	
**No**		141 (89.8%)	416 (89.3%)	557 (89.4%)	
**Yes**		16 (10.2%)	50 (10.7%)	66 (10.6%)	
**Alcohol**	ind				0.325
**N-Miss**		21	52	73	
**No**		123 (90.4%)	364 (87.3%)	487 (88.1%)	
**Yes**		13 (9.6%)	53 (12.7%)	66 (11.9%)	
**Age**	ind				0.002
**Mean (SD)**		65.06 (9.79)	67.62 (8.74)	66.97 (9.08)	
**Range**		33.00–84.00	32.00–88.00	32.00–88.00	
**SSD Q1**	ind				0.013
**N-Miss**		14	34	48	
**Mean (SD)**		6.20 (1.73)	5.74 (1.94)	5.86 (1.90)	
**Range**		1.00–10.00	0.00–10.00	0.00–10.00	
**SSD Q2**	ind				0.098
**N-Miss**		14	34	48	
**Mean (SD)**		6.13 (2.01)	5.79 (2.12)	5.88 (2.10)	
**Range**		0.00–10.00	0.00–10.00	0.00–10.00	
**Martial with**	ind				0.614
**N-Miss**		1	5	6	
**No**		30 (19.2%)	98 (21.1%)	128 (20.6%)	
**Yes**		126 (80.8%)	366 (78.9%)	492 (79.4%)	
**Higher school**	ind				0.926
**N-Miss**		2	7	9	
**No**		118 (76.1%)	350 (75.8%)	468 (75.9%)	
**Yes**		37 (23.9%)	112 (24.2%)	149 (24.1%)	
**Employed**	ind				0.325
**N-Miss**		21	52	73	
**No**		123 (90.4%)	364 (87.3%)	487 (88.1%)	
**Yes**		13 (9.6%)	53 (12.7%)	66 (11.9%)	
**Income**	ind				0.032
**N-Miss**		38	110	148	
**Mean (SD)**		5.15 (2.00)	5.60 (1.94)	5.49 (1.97)	
**Range**		1.00–8.00	1.00–8.00	1.00–8.00	
**Number Children**	ind				0.305
**N-Miss**		12	47	59	

**Mean (SD)**		1.68 (0.98)	1.78 (1.07)	1.75 (1.05)	
**Range**		0.00–5.00	0.00–10.00	0.00–10.00	
**EQ-5D-5L**	ind				0.011
**N-Miss**		2	11	13	
**Mean (SD)**		0.63 (0.19)	0.67 (0.18)	0.66 (0.18)	
**Range**		0.14–0.92	0.05–1.00	0.05–1.00	
**OSSS**	ind				< 0.001
**N-Miss**		1	6	7	
**Mean (SD)**		10.51 (2.21)	11.17 (1.96)	11.00 (2.04)	
**Range**		4.00–14.00	5.00–14.00	4.00–14.00	
**Pre-treatment**	ind				0.880
**N-Miss**		13	41	54	
**Mean (SD)**		2981.18 (4218.51)	3039.62 (3968.95)	3024.91 (4029.56)	
**Range**		11.00–24588.00	18.00–25032.00	11.00–25032.00	
**PHQ-4**	ind				< 0.001
**N-Miss**		12	32	44	
**Mean (SD)**		3.49 (2.96)	2.14 (2.28)	2.48 (2.53)	
**Range**		0.00–12.00	0.00–12.00	0.00–12.00	
**LOT-R opt**	ind				< 0.001
**N-Miss**		5	16	21	
**Mean (SD)**		9.45 (2.42)	10.46 (2.18)	10.21 (2.28)	
**Range**		3.00–12.00	0.00–12.00	0.00–12.00	
**LOT-R pess**	ind				< 0.001
**N-Miss**		6	16	22	
**Mean (SD)**		8.23 (3.06)	9.70 (2.59)	9.33 (2.79)	
**Range**		0.00–12.00	0.00–12.00	0.00–12.00	
**KOOS pain**	ind				< 0.001
**N-Miss**		1	4	5	
**Mean (SD)**		41.90 (15.72)	48.13 (17.28)	46.56 (17.10)	
**Range**		0.00–87.50	0.00–91.67	0.00–91.67	
**KOOS symp**	ind				< 0.001
**N-Miss**		1	3	4	
**Mean (SD)**		47.33 (17.55)	55.73 (19.38)	53.62 (19.28)	
**Range**		3.57–89.29	3.57–100.00	3.57–100.00	
**KOOS ADL**	ind				< 0.001
**N-Miss**		1	3	4	
**Mean (SD)**		50.54 (19.24)	57.06 (17.93)	55.42 (18.47)	
**Range**		1.56–98.53	4.41–95.59	1.56–98.53	
**KOOS sport**	ind				0.076
**N-Miss**		5	20	25	
**Mean (SD)**		16.07 (15.90)	18.95 (17.72)	18.22 (17.31)	
**Range**		0.00–80.00	0.00–90.00	0.00–90.00	
**KOOS QoL**	ind				0.159
**N-Miss**		3	6	9	

**Mean (SD)**		23.50 (14.17)	25.34 (13.96)	24.88 (14.02)	
**Range**		0.00–75.00	0.00–81.25	0.00–81.25	
**Staffelstein**	ind				0.010
**N-Miss**		28	66	94	
**Mean (SD)**		78.33 (12.43)	81.55 (12.37)	80.77 (12.45)	
**Range**		38.00–111.00	36.00–115.00	36.00–115.00	
**WOMAC pain**	ind				< 0.001
**N-Miss**		1	5	6	
**Mean (SD)**		45.80 (17.12)	53.99 (18.29)	51.93 (18.33)	
**Range**		0.00–90.00	0.00–95.00	0.00–95.00	
**ASA**	pre				0.973
**N-Miss**		3	2	5	
**Mean (SD)**		2.24 (0.52)	2.24 (0.55)	2.24 (0.54)	
**Range**		1.00–3.00	1.00–4.00	1.00–4.00	
**SSD Q1**	pre				0.482
**N-Miss**		57	128	185	
**Mean (SD)**		5.73 (1.94)	5.58 (1.82)	5.62 (1.85)	
**Range**		0.00–10.00	0.00–10.00	0.00–10.00	
**TUG**	pre				0.621
**N-Miss**		15	31	46	
**Mean (SD)**		12.38 (4.10)	12.17 (4.52)	12.22 (4.42)	
**Range**		6.00–29.00	6.00–49.00	6.00–49.00	
**Pain rest**	pre				< 0.001
**N-Miss**		19	46	65	
**Mean (SD)**		3.48 (2.44)	2.43 (2.37)	2.68 (2.43)	
**Range**		0.00–9.00	0.00–10.00	0.00–10.00	
**Pain load**	pre				0.016
**N-Miss**		19	47	66	
**Mean (SD)**		5.25 (2.30)	4.64 (2.63)	4.79 (2.56)	
**Range**		0.00–10.00	0.00–10.00	0.00–10.00	
**ISAR**	pre				0.005
**N-Miss**		40	97	137	
**Mean (SD)**		0.58 (0.84)	0.35 (0.72)	0.41 (0.76)	
**Range**		0.00–4.00	0.00–6.00	0.00–6.00	
**PHQ-4**	pre				< 0.001
**N-Miss**		56	124	180	
**Mean (SD)**		3.81 (3.17)	2.03 (2.13)	2.43 (2.52)	
**Range**		0.00–12.00	0.00–12.00	0.00–12.00	
**Height**	surg				0.986
**Mean (SD)**		170.92 (9.71)	170.90 (9.28)	170.91 (9.38)	
**Range**		148.00–198.00	136.00–198.00	136.00–198.00	
**Weight**	surg				0.027
**Mean (SD)**		90.55 (18.46)	86.90 (17.76)	87.81 (18.00)	
**Range**		53.00–150.00	45.00–175.00	45.00–175.00	

**Surg duration**	surg				0.128
**Mean (SD)**		70.17 (34.78)	65.78 (29.85)	66.88 (31.19)	
**Range**		17.00–278.00	23.00–243.00	17.00–278.00	
**Cortisone**	surg				0.881
**No**		4 (2.5%)	13 (2.8%)	17 (2.7%)	
**Yes**		153 (97.5%)	456 (97.2%)	609 (97.3%)	
**Surg tranex**	surg				0.619
**No**		14 (8.9%)	36 (7.7%)	50 (8.0%)	
**Yes**		143 (91.1%)	433 (92.3%)	576 (92.0%)	
**Intub anesthesia**	surg				0.480
**N-Miss**		52	182	234	
**SPA**		43 (41.0%)	129 (44.9%)	172 (43.9%)	
**ITN**		62 (59.0%)	158 (55.1%)	220 (56.1%)	
**Surg tourn**	surg				0.181
**No**		109 (69.4%)	298 (63.5%)	407 (65.0%)	
**Yes**		48 (30.6%)	171 (36.5%)	219 (35.0%)	
**Surg LIA**	surg				0.905
**No**		7 (4.5%)	22 (4.7%)	29 (4.6%)	
**Yes**		150 (95.5%)	447 (95.3%)	597 (95.4%)	
**Surg drain**	surg				0.866
**No**		154 (98.1%)	461 (98.3%)	615 (98.2%)	
**Yes**		3 (1.9%)	8 (1.7%)	11 (1.8%)	
**Walking dist**	post				0.188
**N-Miss**		89	306	395	
**Mean (SD)**		1.94 (0.45)	2.04 (0.52)	2.01 (0.50)	
**Range**		1.00–3.00	1.00–4.00	1.00–4.00	
**SSD Q1**	post				0.534
**N-Miss**		13	38	51	
**Mean (SD)**		4.85 (2.32)	4.98 (2.26)	4.95 (2.27)	
**Range**		0.00–9.00	0.00–10.00	0.00–10.00	
**SSD Q2**	post				0.922
**N-Miss**		14	42	56	
**Mean (SD)**		4.98 (2.65)	5.01 (2.42)	5.00 (2.48)	
**Range**		0.00–10.00	0.00–10.00	0.00–10.00	
**TUG**	post				0.023
**N-Miss**		25	87	112	
**Mean (SD)**		18.22 (8.26)	16.70 (5.91)	17.09 (6.62)	
**Range**		7.00–88.00	5.00–53.00	5.00–88.00	
**Pain rest**	post				< 0.001
**N-Miss**		21	73	94	
**Mean (SD)**		2.62 (1.90)	1.92 (1.72)	2.10 (1.79)	
**Range**		0.00–8.00	0.00–8.00	0.00–8.00	
**Pain load**	post				0.002
**N-Miss**		21	73	94	

Mean (SD)		3.89 (1.95)	3.32 (1.80)	3.46 (1.85)	
Range		0.00–9.00	0.00–8.00	0.00–9.00	
**EQ-5D-5L**	post				0.022
N-Miss		9	24	33	
Mean (SD)		0.76 (0.17)	0.80 (0.15)	0.79 (0.15)	
Range		0.17–1.00	0.26–1.00	0.17–1.00	
**LOS**	post				0.637
Mean (SD)		5.46 (2.61)	5.35 (2.53)	5.38 (2.55)	
Range		2.00–24.00	2.00–37.00	2.00–37.00	
**PHQ-4**	post				< 0.001
N-Miss		12	33	45	
Mean (SD)		2.71 (2.79)	1.71 (2.10)	1.96 (2.33)	
Range		0.00–12.00	0.00–12.00	0.00–12.00	
**Staffelstein**	post				0.191
N-Miss		8	15	23	
Mean (SD)		76.10 (14.54)	77.97 (15.36)	77.51 (15.17)	
Range		29.00–110.00	25.00–119.00	25.00–119.00	
**EQ-5D-5L**	rehab				< 0.001
N-Miss		57	187	244	
Mean (SD)		0.77 (0.14)	0.84 (0.11)	0.82 (0.12)	
Range		0.36–1.00	0.21–1.00	0.21–1.00	
**TUG**	rehab				0.752
N-Miss		98	313	411	
Mean (SD)		10.86 (3.68)	11.08 (4.63)	11.02 (4.39)	
Range		5.00–22.00	5.00–44.00	5.00–44.00	
**Pain rest**	rehab				0.001
N-Miss		93	301	394	
Mean (SD)		2.28 (1.85)	1.50 (1.51)	1.72 (1.65)	
Range		0.00–8.00	0.00–6.00	0.00–8.00	
**Pain load**	rehab				0.002
N-Miss		94	301	395	
Mean (SD)		3.68 (1.94)	2.86 (1.71)	3.09 (1.81)	
Range		0.00–8.00	0.00–8.00	0.00–8.00	
**PHQ-4**	rehab				< 0.001
N-Miss		57	188	245	
Mean (SD)		2.49 (2.12)	1.04 (1.73)	1.42 (1.95)	
Range		0.00–8.00	0.00–12.00	0.00–12.00	
**rehab duration**	rehab				0.498
N-Miss		56	178	234	
Mean (SD)		23.07 (5.24)	21.84 (4.26)	22.16 (4.56)	
Range		12.00–52.00	4.00–42.00	4.00 - 52.00	
**Staffelstein**	rehab				< 0.001
N-Miss		86	231	317	
Mean (SD)		85.04 (12.36)	92.43 (13.21)	90.73 (13.37)	
Range		48.00–109.00	47.00–120.00	47.00–120.00	

**SSD Q1**	3mo				< 0.001
**N-Miss**		15	49	64	
**Mean (SD)**		4.21 (1.88)	2.72 (1.97)	3.10 (2.05)	
**Range**		0.00–8.00	0.00–9.00	0.00–9.00	
**EQ-5D-5L**	3mo				< 0.001
**N-Miss**		12	41	53	
**Mean (SD)**		0.74 (0.18)	0.87 (0.11)	0.84 (0.14)	
**Range**		0.07–1.00	0.26–1.00	0.07–1.00	
**KOOS pain**	3mo				< 0.001
**N-Miss**		11	31	42	
**Mean (SD)**		55.16 (17.13)	75.76 (15.37)	70.61 (18.16)	
**Range**		5.56–100.00	36.11–100.00	5.56–100.00	
**KOOS symp**	3mo				< 0.001
**N-Miss**		11	29	40	
**Mean (SD)**		54.06 (17.52)	72.40 (15.05)	67.83 (17.58)	
**Range**		17.86–96.43	28.57–100.00	17.86–100.00	
**KOOS ADL**	3mo				< 0.001
**N-Miss**		11	31	42	
**Mean (SD)**		60.62 (16.21)	79.64 (13.60)	74.88 (16.49)	
**Range**		11.76–95.59	26.56–100.00	11.76–100.00	
**KOOS sport**	3mo				< 0.001
**N-Miss**		29	94	123	
**Mean (SD)**		29.41 (21.90)	49.59 (25.18)	44.46 (25.91)	
**Range**		0.00–80.00	0.00–100.00	0.00–100.00	
**KOOS QoL**	3mo				< 0.001
**N-Miss**		9	32	41	
**Mean (SD)**		36.53 (19.02)	56.66 (20.86)	51.56 (22.19)	
**Range**		0.00–93.75	0.00–100.00	0.00–100.00	
**WOMAC pain**	3mo				< 0.001
**N-Miss**		11	31	42	
**Mean (SD)**		59.90 (17.48)	79.86 (14.38)	74.87 (17.49)	
**Range**		10.00–100.00	40.00–100.00	10.00–100.00	
**PHQ-4**	3mo				< 0.001
**N-Miss**		20	64	84	
**Mean (SD)**		2.97 (2.86)	1.09 (1.90)	1.57 (2.33)	
**Range**		0.00–12.00	0.00–12.00	0.00–12.00	
**SSD Q1**	6mo				< 0.001
**N-Miss**		22	51	73	
**Mean (SD)**		4.16 (1.95)	2.24 (1.85)	2.71 (2.05)	
**Range**		0.00–9.00	0.00–10.00	0.00–10.00	
**SSD Q2**	6mo				< 0.001
**N-Miss**		23	56	79	
**Mean (SD)**		4.13 (2.14)	2.08 (1.93)	2.59 (2.17)	
**Range**		0.00–9.00	0.00–10.00	0.00–10.00	
**EQ-5D-5L**	6mo				< 0.001
**N-Miss**		20	36	56	

**Mean (SD)**		0.75 (0.18)	0.90 (0.11)	0.86 (0.14)	
**Range**		0.04–1.00	0.14–1.00	0.04–1.00	
**PHQ-4**	6mo				< 0.001
**N-Miss**		27	62	89	
**Mean (SD)**		2.88 (2.66)	0.93 (1.64)	1.40 (2.11)	
**Range**		0.00–11.00	0.00–12.00	0.00–12.00	
**KOOS pain**	6mo				< 0.001
**N-Miss**		16	19	35	
**Mean (SD)**		58.34 (16.85)	82.80 (13.86)	76.96 (17.95)	
**Range**		16.67–100.00	33.33–100.00	16.67–100.00	
**KOOS symp**	6mo				< 0.001
**N-Miss**		17	20	37	
**Mean (SD)**		58.21 (14.42)	78.81 (14.61)	73.91 (17.00)	
**Range**		14.29–89.29	28.57–100.00	14.29–100.00	
**KOOS ADL**	6mo				< 0.001
**N-Miss**		19	21	40	
**Mean (SD)**		62.72 (15.65)	84.16 (12.55)	79.11 (16.15)	
**Range**		20.59–92.65	35.29–100.00	20.59–100.00	
**KOOS sport**	6mo				< 0.001
**N-Miss**		28	60	88	
**Mean (SD)**		32.29 (21.60)	58.92 (23.32)	52.53 (25.57)	
**Range**		0.00–85.00	0.00–100.00	0.00–100.00	
**KOOS QoL**	6mo				< 0.001
**N-Miss**		16	22	38	
**Mean (SD)**		40.65 (19.52)	65.57 (20.48)	59.59 (22.87)	
**Range**		0.00–91.67	0.00–100.00	0.00–100.00	
**WOMAC pain**	6mo				< 0.001
**N-Miss**		16	19	35	
**Mean (SD)**		63.05 (17.17)	86.01 (12.99)	80.53 (17.16)	
**Range**		20.00–100.00	25.00–100.00	20.00–100.00	
**SSD Q1**	1y				< 0.001
**N-Miss**		**11**	**23**	**34**	
Mean (SD)		3.95 (2.07)	1.84 (1.81)	2.36 (2.09)	
Range		0.00 - 10.00	0.00 - 9.00	0.00 - 10.00	
**SSD Q2**	1y				< 0.001
N-Miss		10	23	33	
Mean (SD)		4.03 (2.28)	1.64 (1.80)	2.24 (2.19)	
Range		0.00 - 10.00	0.00 - 10.00	0.00 - 10.00	
**EQ-5D-5L**	1y				< 0.001
N-Miss		8	20	28	
Mean (SD)		0.75 (0.18)	0.93 (0.10)	0.89 (0.14)	
Range		0.09 - 1.00	0.26 - 1.00	0.09 - 1.00	
**PHQ-4**	1y				< 0.001
N-Miss		13	41	54	
Mean (SD)		2.74 (2.99)	0.80 (1.49)	1.29 (2.15)	
Range		0.00 - 12.00	0.00 - 10.00	0.00 - 12.00	
**KOOS symp**	1y				< 0.001
N-Miss		3	3	6	
Mean (SD)		61.51 (15.61)	84.79 (12.11)	79.01 (16.48)	

**Range**		14.29 - 95.83	42.86 - 100.00	14.29 - 100.00	
**KOOS ADL**	1y				< 0.001
**N-Miss**		0	2	2	
**Mean (SD)**		62.81 (16.35)	90.22 (9.15)	83.32 (16.47)	
**Range**		6.67 - 100.00	45.59 - 100.00	6.67 - 100.00	
**KOOS sport**	1y				< 0.001
**N-Miss**		14	56	70	
**Mean (SD)**		35.57 (24.58)	67.68 (21.08)	59.42 (26.11)	
**Range**		0.00 - 90.00	0.00 - 100.00	0.00 - 100.00	
**KOOS Qol**	1y				< 0.001
**N-Miss**		2	6	8	
**Mean (SD)**		42.31 (18.54)	72.95 (18.71)	65.26 (22.90)	
**Range**		0.00 - 87.50	18.75 - 100.00	0.00 - 100.00	

Table S2: Details for the prediction model based on preoperative data, non-standardized and standardized, using LASSO min

**Using non-standardized data**				
(Intercept)	Alcohol, ind	Age, ind	Martial with, ind	Higher school, pre
-1.238	0.040	0.014	-0.069	-0.018
Number children, ind	OSSS, ind	LOT-R opt, ind	LOT-R pess. ind	KOOS symp, ind
0.019	0.037	0.031	0.08	0.005
KOOS QoL, ind	WOMAC pain, ind	SSD Q1, pre	TUG, pre	VAS rest, pre
-0.003	0.003	0.05	-0.003	-0.09
ISAR, pre	PHQ-4, pre			
-0.05	-0.1			
**Using standardized data**				
(Intercept)	Alcohol, ind	Age, ind	Martial with, ind	Higher school, pre
1.065	0.013	0.133	-0.030	-0.008
Number children, ind	OSSS, ind	LOT-R opt, ind	LOT-R pess. ind	KOOS symp, ind
0.021	0.078	0.072	0.2	0.1
KOOS QoL, ind	WOMAC pain, ind	SSD Q1, pre	TUG, pre	VAS rest, pre
-0.04	0.05	0.09	-0.02	-0.2
ISAR, pre	PHQ-4, pre			
-0.04	-0.4			

Table S3: Details for the prediction model based on data up to 6 month after surgery, non-standardized and standardized, using LASSO min

**Using non-standardized data**				
(Intercept)	Number children, ind	LOT-R pess, ind	KOOS QoL, ind	SSD Q1, pre
-6.01	0.02	0.04	-0.004	0.01
TUG, pre	VAS rest, pre	PHQ-4, pre	Cortisone, surg	Walking dist, post
-0.0002	-0.05	-0.08	0.2	0.09
SSD Q1, post	VAS load, post	SSD Q2, rehab	TUG, rehab	VAS rest, rehab
0.07	0.01	-0.02	0.003	-0.01
VAS load, rehab	Staffelstein, rehab	SSD Q2, 3 mo	KOOS pain, 3 mo	KOOS ADL, 3 mo
-0.04	0.01	-0.01	0.01	0.01
WOMAC pain, 3 mo	EQ-5D-5L, 6 mo	PHQ-4, 6 mo	KOOS symp, 6 mo	KOOS ADL, 6 mo
0.01	0.46	-0.05	0.02	0.01
KOOS sport, 6 mo	WOMAC pain, 6 mo			
0.01	0.02			
**Using standardized data**				
(Intercept)	Number children, ind	LOT-R pess, ind	KOOS QoL, ind	SSD Q1, pre
1.29	0.02	0.12	-0.05	0.01
TUG, pre	VAS rest, pre	PHQ-4, pre	Cortisone, surg	Walking dist, post
-0.0009	-0.13	-0.21	0.03	0.05
SSD Q1, post	VAS load, post	SSD Q2, rehab	TUG, rehab	VAS rest, rehab
0.18	0.01	-0.05	0.01	-0.01
VAS load, rehab	Staffelstein, rehab	SSD Q2, 3 mo	KOOS pain, 3 mo	KOOS ADL, 3 mo
-0.08	0.14	-0.02	0.09	0.1
WOMAC pain, 3 mo	EQ-5D-5L, 6 mo	PHQ-4, 6 mo	KOOS symp, 6 mo	KOOS ADL, 6 mo
0.21	0.07	-0.11	0.36	0.15
KOOS sport, 6 mo	WOMAC pain, 6 mo			
0.15	0.32			
